# 

**Published:** 1892-01

**Authors:** 


### Communications.

### POST GRADUATE DENTAL ASSOCIATION.

The Post Graduate Dental Association of the United States will hold its annual meetiug April 29th and 30th next at the Leland Hotel, Chicago.

Dr. W. C. Barrett, of Buffalo, N. Y.; Drs. T. W. Brophy, Louis Ottofy and others, of Chicago, will present essays and addresses. An interesting programme has been prepared, and a good attendance is expected.

All members of the profession are invited.

Graduates of recognized dental colleges may become members by paying membership fee, $1.00; and dues for one year in advance, $1.00.

T. S. TENNEY, *Sec'y.*,
96 State St., Chicago.

R. B. FULLER, *Pres.*

---

### Editorial, Etc.

### To Readers and Correspondents.

Communications intended for publication, and exchange journals and papers, should be addressed to the Editor of The American Journal of Dental Science, No. 845 N. Eutaw St. Baltimore, Md. All letters relating to the business of the Journal, or containing cash remittances, to be directed to Messrs. Snowden & Cowman, No. 9 W. Fayette Street, Baltimore, Md. Articles for publication should be received by the Editor at least two weeks previous to the first of the month on which the number in which it is designed for them to appear, is issued.

☞ The American Journal of Dental Science will contain fifty pages of reading matter in each number, making a volume of about Six Hundred pages,

EXCLUSIVE OF ADVERTISEMENTS

